# Grape Seed Procyanidin Extract Improves Insulin Production but Enhances Bax Protein Expression in Cafeteria-Treated Male Rats

**DOI:** 10.1155/2013/875314

**Published:** 2013-04-18

**Authors:** Lídia Cedó, Anna Castell-Auví, Victor Pallarès, Mayte Blay, Anna Ardévol, Montserrat Pinent

**Affiliations:** Nutrigenomics Research Group, Departament de Bioquímica i Biotecnologia, Universitat Rovira i Virgili, Marcel*·*lí Domingo s/n, 43007 Tarragona, Spain

## Abstract

In a previous study, the administration of a grape seed procyanidin extract (GSPE) in female Wistar rats improved insulin resistance, reduced insulin production, and modulated apoptosis biomarkers in the pancreas. Considering that pharmacokinetic and pharmacodynamic parameters in females are different from these parameters in males, the aim of the present study was to evaluate the effects of GSPE on male Wistar cafeteria-induced obese rats. The results have confirmed that the cafeteria model is a robust model mimicking a prediabetic state, as these rats display insulin resistance, increased insulin synthesis and secretion, and increased apoptosis in the pancreas. In addition, GSPE treatment (25 mg/kg of GSPE for 21 days) in male rats improves insulin resistance and counteracts the cafeteria-induced effects on insulin synthesis. However, the administration of the extract enhances the cafeteria-induced increase in Bax protein levels, suggesting increased apoptosis. This result contradicts previous results from cafeteria-fed female rats, in which GSPE seemed to counteract the increased apoptosis induced by the cafeteria diet.

## 1. Introduction 

Procyanidins are the second most abundant natural phenolic after lignin, and they are widely distributed in fruits, berries, beans, nuts, cocoa, and wine [1]. They are potent antioxidants that possess biological properties that may protect against cardiovascular diseases [1]. They participate in glucose homeostasis [2] and modulate insulin synthesis, secretion, and degradation [3]. Moreover, changes in *β*-cell insulin production may also be due to variations in the number of insulin-producing cells. *β*-cell mass adapts to increased metabolic demands caused, for example, by obesity, pregnancy, or insulin resistance. However, when *β*-cells are unable to compensate for increased insulin demand, there is a decrease in *β*-cell mass characteristic of the onset of type 2 diabetes mellitus (T2DM) [4]. Procyanidins modulate apoptotic and proliferation processes, mainly reported in cancerous cell lines [5]. Moreover, they protect cells from diverse drug- or chemical-induced toxic assaults by decreasing apoptosis and inducing cell growth [5]. However, there is little information available regarding their effects on *β*-cells. Other studies by our research group have reported that procyanidins modulate proliferation and apoptosis of the pancreatic *β*-cell line INS-1E under altered conditions [6]. Procyanidins also alter the protein and/or gene expression of factors involved in apoptosis in Zucker fatty rats [7].

Obesity has become a worldwide problem, leading to an explosion of obesity-related health issues [8]. Obese individuals develop resistance to the cellular actions of insulin, a key etiological factor for T2DM, which is also becoming an epidemic [9]. T2DM is characterised by peripheral insulin resistance as well as pancreatic *β*-cell dysfunction and decreased *β*-cell mass [10]. It involves a combination of genetic and environmental or lifestyle factors. These lifestyle changes, involving high-energy diets and reduced physical activity, are linked to the pandemics of obesity and T2DM [11]. Given the high prevalence of the disease, obtaining knowledge about natural compounds with potential beneficial effects on glucose homeostasis is of great interest. 

Several animal models have been used to study obesity, including both genetic and diet-induced obesity models. However, the cafeteria diet is a more robust model to reproduce the diet in Western society [12]. In a previous study, we analysed the effects of procyanidins in an insulin resistance model induced by cafeteria diet administration in female Wistar rats. We also considered the effects of the cafeteria diet on insulin production and apoptosis in the pancreas. The study showed that the cafeteria diet increased insulin production as well as activated apoptosis biomarkers [13]. Furthermore, procyanidin administration caused a reduction in the Homeostasis Model Assessment for Insulin Resistance (HOMA-IR) index, suggesting improved insulin resistance [14]. Moreover, in the pancreas, procyanidins caused a decrease in insulin production [15] and modulated pro- and antiapoptosis markers [6].

The pharmacokinetics and pharmacodynamics in females are different from the same parameters in males because of the female's unique anatomy and physiology [16, 17]. Thus, the aim of the present study was to evaluate the effects of GSPE in male Wistar cafeteria-induced obese rats and to compare the results on insulin synthesis, apoptosis, and proliferation in the pancreas with those observed in the previous study of female rats.

## 2. Materials and Methods

### 2.1. GSPE

The procyanidin extract was derived from grape seed and contained the following: catechin (58 *μ*mol/g), epicatechin (52 *μ*mol/g), epigallocatechin (5.50 *μ*mol/g), epicatechin gallate (89 *μ*mol/g), epigallocatechin gallate (1.40 *μ*mol/g), dimeric procyanidins (250 *μ*mol/g), trimeric procyanidins (15.68 *μ*mol/g), tetrameric procyanidins (8.8 *μ*mol/g), pentameric procyanidins (0.73 *μ*mol/g), and hexameric procyanidins (0.38 *μ*mol/g) [18].

### 2.2. Animal Experimental Procedures

Wistar male rats weighting between 250–330 g were purchased from Charles River Laboratories (Barcelona, Spain) and housed in animal quarters at 22°C with a 12 h light/dark cycle. After 1 week in quarantine, the animals were divided in two groups, a diet-control group (7 animals) fed a standard diet (Panlab A03) and a cafeteria group (21 animals) fed a cafeteria diet (bacon, biscuits with pâté, biscuits with cheese, muffins, carrots, and milk with sugar) in addition to standard chow and water. Every day at 9 AM, food was withdrawn, and it was replaced at 6 PM. Obesity was induced in the animals on the cafeteria diet for 52 days. Afterwards, the diet-control group and 7 animals from the cafeteria-fed group were sacrificed, as a reference for the state of the animals before the beginning of treatment. The rest of the cafeteria-fed rats were divided in two subgroups (7 animals/group). These two groups were the (i) cafeteria group: rats treated with a vehicle (gum arabic 5% w/v) and the (ii) GSPE-treated group: rats treated with 25 mg of GSPE/kg of body weight (bw) per day. The treatment was administrated every evening for 21 days before the replacement of the food. Three days before the end of the treatment and after 8 h of fasting, blood was collected from the tails of the rats to measure glucose and insulin levels. At the end of the treatment regimen and after 3 h of fasting, the animals were anesthetised using sodium pentobarbital (50 mg/kg of bw, Sigma-Aldrich, St. Louis, MO) and sacrificed by abdominal aorta exsanguination. The pancreas was isolated from all of the animals, frozen immediately in liquid nitrogen, and stored at −80°C until analysis. All of the procedures were approved by the Experimental Animals Ethics Committee of the Universitat Rovira i Virgili. 

### 2.3. Plasmatic and Pancreatic Measurements

Insulin plasma levels were assayed using an ELISA method following the manufacturer's instructions (Mercodia, Uppsala, Sweden). Glucose plasma levels were determined using an enzymatic colorimetric kit (QCA, Amposta, Spain). 

The HOMA-IR and HOMA-*β* were calculated using the fasting values of glucose and insulin and the following formulas:

(1) HOMA-IR=insulin  (μU/mL)  ×  glucose  (mM)  22.5,HOMA-β=20×insulin  (μU/mL)  glucose  (mM)−3.5.

Triglycerides (TAG) and nonesterified fatty acids (NEFAs) from the pancreas were extracted by homogenising the tissue with PBS containing 0.1% triton X-100 (Sigma-Aldrich, St. Louis, MO), and their concentrations were determined using enzymatic colorimetric kits (QCA, Amposta, Spain for TAG and Wako chemicals GmbH, Neuss, Germany for NEFAs).

The pancreas was homogenised with six volumes of PBS containing 50 mM EDTA at pH 7.4 and centrifuged at 3000 g for 5 min at 4°C. Reactive oxygen species (ROS) in the supernatants were quantified using 20 *μ*M DCFH-DA (2′,7′-dichlorofluorescin diacetate) (Sigma-Aldrich, St. Louis, MO), and the fluorescence was measured after 50 minutes at 37°C at *λ*_ex_ = 485 nm and *λ*_em_ = 530 nm. The values were normalised to the protein content, which was analysed by the Bradford method [19].

### 2.4. Quantitative Real-Time PCR

Total RNA from the pancreas was extracted using the RNeasy Mini Kit (Qiagen, Barcelona, Spain). cDNA was generated with the High-Capacity cDNA Reverse Transcription Kit (Applied Biosystems, Madrid, Spain) and was subjected to quantitative Real-Time PCR amplification using the TaqMan Master Mix (Applied Biosystems, Madrid, Spain). Specific TaqMan probes (Applied Biosystems, Madrid, Spain) were used for each gene: Rn99999125_m1 for *Bcl2*, Rn01480160_g1 for *Bax*, Rn01492401_m1 for *Ccnd2* (Cyclin D2), Rn01774648_g1 for *Ins*, and Rn00565544_m1 for *Cpe*. *Actb* was used as the reference gene (Rn00667869_m1). Reactions were run on a quantitative RT-PCR 7300 system (Applied Biosystems, Madrid, Spain) according to the manufacturer's instructions. The relative mRNA expression levels were calculated using the ΔΔCt method.

### 2.5. Western Blot

The protein levels of Bax and Bcl-2 were quantified by Western Blot as previously described [13]. Primary antibodies were purchased from Cell Signalling Technology (Beverly, MA). 25 *μ*g of protein was loaded onto the gel, and the antibody dilution was 1 : 1500 for Bax and Bcl-2. After incubation with peroxidase-conjugated monoclonal anti-rabbit secondary antibody (Sigma-Aldrich, Madrid, Spain) at a 1 : 10000 dilution, immunoreactive proteins were visualised with the ECL Plus Western Blotting Detection System (GE Healthcare, Buckinghamshire, UK). Chemiluminescence and densitometric analysis of the immunoblots was performed using ImageJ 1.44p software, and all proteins were quantified relative to the loading control.

### 2.6. Calculations and Statistical Analysis

The results are expressed as the mean ± SEM. Effects were assessed using Student's *t*-test. All calculations were performed with SPSS software v19.

## 3. Results

### 3.1. Cafeteria Diet Increases Insulin Production in the Pancreas and GSPE Treatment Counteracts This Diet

We first examined the effects of the cafeteria diet on pancreatic insulin production after 52 days of diet administration. Insulin and glucose plasma levels were quantified at day 49, and the cafeteria diet-fed rats showed significantly higher plasma insulin levels and no changes in glucose levels (Table 1). The HOMA-IR index indicated that the cafeteria-fed animals had peripheral insulin resistance, and their HOMA-*β* index tended to increase (Table 1). Therefore, there was a tendency to increase pancreatic functionality response to glucose in order to counteract peripheral insulin resistance. The increased plasma insulin levels agree with an increase in the insulin gene expression, as well as with increased gene expression of carboxypeptidase E (Cpe) (Table 2).

After induction of obesity via the cafeteria diet, rats were treated with 25 mg/kg of bw GSPE for 21 days, concomitantly with cafeteria diet administration. The animals treated with the procyanidin extract had lower insulinemia and decreased HOMA-IR and HOMA-*β* indexes (Table 1), counteracting the effects observed in the cafeteria-fed rats. Moreover, insulin gene expression tended to decrease in these rats, and decreased expression of Cpe was also observed (Table 3).

### 3.2. Effects of Cafeteria Diet and GSPE on Apoptosis Biomarkers

To examine the effects of the cafeteria diet and GSPE on apoptosis and proliferation in the pancreas, several markers were analysed at the gene and protein level.

**Figure 1:**
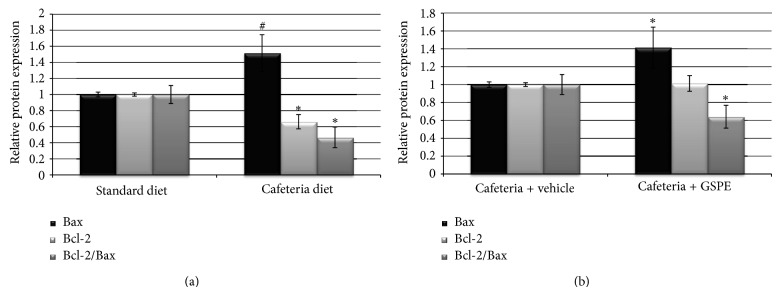
Protein expression of the apoptosis markers Bcl-2 and Bax and the calculated ratio of Bcl-2/Bax in: (a) standard-diet-fed rats and cafeteria-diet-fed rats and (b) in GSPE-treated rats and vehicle-treated rats assessed by Western Blot. Data are shown as the mean ± SEM. ∗*P* ≤ 0.05 and ^#^ *P* ≤ 0.1.

The cafeteria-fed rats showed a decrease in the antiapoptotic marker Bcl-2 at both the gene (Table 2) and protein levels (Figure 1(a)). For the pro-apoptotic marker Bax, the mRNA levels of this gene were not significantly altered (Table 2), but we did observe an increase in the protein levels of Bax in the cafeteria-diet-fed group (Figure 1(a)). Therefore, the ratio of Bcl-2/Bax was reduced both at the gene and protein levels, suggesting an increase in apoptosis in the pancreas (Table 2 and Figure 1(a)). 

The administration of GSPE had no effect on Bcl-2 and Bax at gene expression compared to the cafeteria diet (Table 3) and on Bcl-2 at protein expression (Figure 1(b)). In contrast, Bax protein levels were increased by the GSPE treatment, enhancing the effects observed in the cafeteria-fed rats (Figure 1(b)). The ratio of Bcl-2/Bax was significantly reduced at protein level (Figure 1(b)) compared to the cafeteria-fed animals.

Finally, we also analysed Cyclin D2, a proliferation marker, but no changes were observed in the cafeteria-fed animals or in the GSPE-treated rats (Table 2).

### 3.3. GSPE Treatment Avoids the Increase of TAG in the Pancreas Induced by Cafeteria Diet

**Figure 2:**
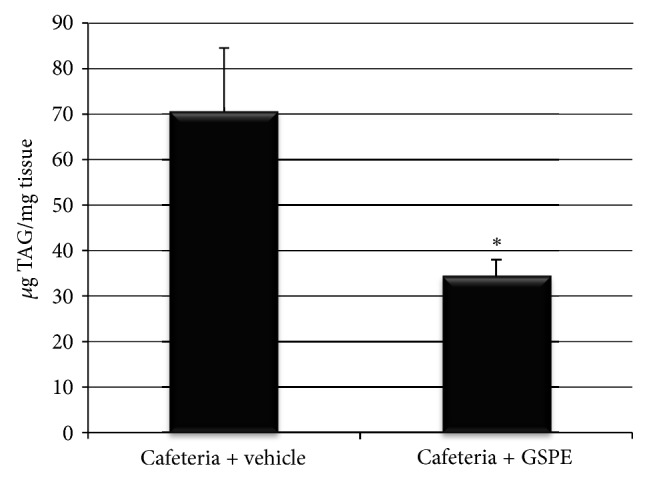
TAG content in the pancreas of cafeteria-fed rats treated with GSPE or vehicle, expressed as *μ*g/mg of pancreatic tissue. Data are shown as the mean ± SEM. ∗*P* ≤ 0.05.

Pancreas malfunction is in part due to the accrual of TAG in its cells. To measure it, we examined the TAG content in this tissue and found that TAG triplicated its levels in the pancreas of cafeteria-fed rats compared to the standard-diet-fed rats (33.15 ± 2.7 versus 11.42 ± 0.3 *μ*g TAG/mg tissue, *P* ≤ 0.01). After 21 days of treatment, the TAG contents in the pancreas increased, due to cafeteria diet, but GSPE avoided this (Figure 2). 

In contrast, the content of NEFAs was not modified neither in the cafeteria group compared to the standard-diet-fed rats (3.37 ± 0.9 versus 4.40 ± 0.4 *μ*g NEFA/mg tissue) nor in the GSPE-treated rats compared to the vehicle-treated rats (2.43 ± 0.3 versus 10.29 ± 4.6 *μ*g NEFA/mg tissue). 

**Figure 3:**
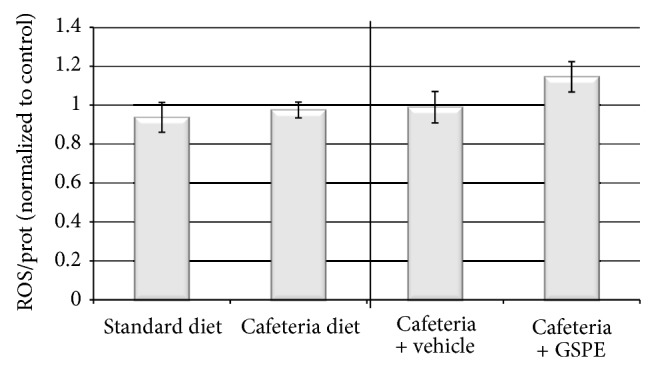
ROS content in the pancreas, expressed as fluorescence arbitrary units/(mg/mL) of protein and normalised to the respective control group. Data are shown as the mean ± SEM.

ROS content in the pancreas was also analysed, and no significant differences were observed in either the cafeteria-fed rats or in the GSPE-treated animals (Figure 3).

## 4. Discussion

This study was designed to examine the effects of the cafeteria diet on insulin production in male Wistar rats by evaluating pancreas functionality, apoptosis, and proliferation. We have also evaluated the effects of procyanidins on these processes, since procyanidins were shown to have positive effects on glucose metabolism under conditions of slightly disrupted homeostasis [2].

We had previously shown that 17 weeks of a cafeteria diet led to insulin resistance, high plasma insulin levels, and increased insulin synthesis and secretion in female Wistar rats [13]. It has been reported that female rats are more sensitive to cafeteria-induced obesity than males [20, 21]. However, with respect to insulin resistance, we now show that 52 days of cafeteria diet (nearly 7 and a half weeks) administrated to male Wistar rats confirms the effects observed in the previous study of female rats. In the males, we have observed high insulin plasma levels and elevated HOMA-IR index, indicating peripheral insulin resistance. Additionally, we have seen an elevated HOMA-*β* index, which indicates an increase in pancreatic functionality in terms of glucose response to counteract peripheral insulin resistance [22]. We have also found an increase in the expression of the insulin gene in the cafeteria-fed rats and an increase in the gene expression of Cpe. Cpe is the enzyme thought to be involved in the cleavage of proinsulin, which results in insulin and C-peptide molecules [23]. Therefore, the pancreas of cafeteria diet-fed rats is still functional, and it tries to counteract peripheral insulin resistance despite the increased lipid accumulation in the pancreas. 

Increased deposits of fat are associated with obesity and lead to an increase in free fatty acids (FFAs). The induction of apoptosis in *in vivo* high-fat diet models and *in vitro* models of FFA-induced apoptosis is important evidence for *β*-cell lipotoxicity [24]. In mice, administration of a high-fat diet for 12 weeks led to increased *β*-cell mass, despite showing an increase in *β*-cell apoptosis [25]. In our study, we have found a decrease in the Bcl-2/Bax ratio both at the gene and protein levels in the pancreas of the cafeteria-fed rats compared to the standard chow-fed rats which also suggest an increase in apoptosis in the cafeteria-fed animals. Palmitate was reported to induce *β*-cell endoplasmic reticulum stress and death mediated by Cpe degradation [26, 27]. Thus we have checked the expression levels of this gene. We have found that Cpe is not decreased but increased by the cafeteria diet, suggesting that the apoptosis observed in the cafeteria-fed rats is not mediated by lipotoxicity. In fact, the levels of NEFA in the pancreas are not altered in the cafeteria-fed rats when compared to the standard-diet-fed rats.

The results from the apoptosis markers are in accordance with the data obtained in the previous study which evaluated the cafeteria diet in Wistar female rats [13]. We have also analyzed the expression of the proliferation marker Cyclin D2 and found no changes due to the cafeteria treatment suggesting that at the time of the analysis, *β*-cell mass had likely already increased. This also agrees with the pervious results in females [6], as well as with those found in rats fed high fat diets, in which no changes in Ki67 (a proliferation marker) expression were observed [25]. 

Taken together, the data suggests that the effects of the cafeteria diet on insulin synthesis, secretion, and apoptosis are not influenced by gender or treatment duration. 

Once the effects of the cafeteria have been established, we have analyzed the effects of a GSPE treatment on the cafeteria-fed animals. After 52 days of cafeteria diet administration, male Wistar rats have been treated with 25 mg/kg of GSPE for 21 days concomitant with the cafeteria diet. The GSPE treatment has decreased insulin production, plasma insulin levels, and pancreatic insulin Cpe gene expression compared to the cafeteria-vehicle-fed rats. Moreover, the decreased insulin production could be at least in part explained through GSPE's lipid-lowering effect, since the triglyceride content has also been reduced in the pancreas of GSPE-treated rats. Previously, we showed that 25 mg of GSPE/kg of bw administered to female Wistar rats for 30 days resulted in decreased insulin production and reduced triglyceride content in the pancreas, likely via decreased fatty acid synthesis and increased *β*-oxidation [15]. Despite this lower insulinemia, GSPE improved glycemia in female Wistar cafeteria-fed rats acting peripherally on adipose tissue [14]. Present results reinforce the effects of GSPE decreasing insulin production and ameliorating lipid accumulation in cafeteria-fed rats and support that these effects are not gender dependent. 

GSPE has counteracted the effects of the cafeteria diet reducing the accumulation of triglycerides in the pancreas but has not counteracted the cafeteria-diet effects on the apoptosis markers. Instead, GSPE-treated rats have shown an increase in Bax protein levels and a decreased ratio of Bcl-2/Bax, suggesting an enhancement in the apoptosis. Therefore, the lipid-lowering effects of GSPE do not involve a reduction in apoptosis in the pancreas of cafeteria-fed male rats. Actually, *in vitro* GSPE does not modulate fatty acid-induced apoptosis [6]. In fact, GSPE increases glucose uptake in *β*-cells under high-glucose conditions and impairs mitochondrial and cellular membrane potentials [3]. Moreover, GSPE is reported to enhance the pro-apoptotic effects of high glucose *in vitro *[6]. Therefore, the enhanced apoptosis observed in the GSPE-treated rats could be due to increased glucose uptake in the *β*-cells that potentiate glucotoxicity. ROS is one of the players in glucose-induced apoptosis in *β*-cells; ROS is increased as a consequence of chronically increased glucose metabolism. *β*-cells have relatively low expression of antioxidant enzymes and are more sensitive to ROS attack when they are exposed to oxidative stress [28]. However, the levels of ROS in the pancreas have not been modified by the cafeteria diet or by GSPE treatment, suggesting that glucose toxicity could be mediated by another mechanism. 

The apoptosis marker results conflict with those in the previous study of female Wistar rats, which showed that 25 and 50 mg/kg of GSPE seemed to counteract the deleterious effects of the cafeteria diet by inhibiting the down-regulation of Bcl-2 protein expression after 10 and 30 days of treatment [6]. In addition, 50 mg/kg bw of GSPE also counteracted the decrease in the Bcl-2/Bax ratio at the protein level after 10 days of administration. However, no GSPE effects were observed with respect to the ratio of Bcl-2/Bax gene expression at any dose or treatment duration [6]. Therefore, the modulation of apoptosis biomarkers by GSPE in cafeteria-fed rats is clearly dependent on the dose and treatment period; these effects may also be dependent on gender.

In conclusion, the present study has confirmed that the cafeteria model is a suitable reproduction of the prediabetic state. This model induces an insulin resistance state, shows increased insulin synthesis and secretion, and exhibits increased apoptosis in the pancreas. Moreover, GSPE treatment in male rats treated with 25 mg/kg of GSPE for 21 days improves the insulin resistance state and counteracts the cafeteria-induced effects on insulin synthesis. However, procyanidins enhance the elevated levels of Bax, a pro-apoptotic protein observed in the cafeteria-fed rats, potentially suggesting an increase in apoptosis. This result indicates that the effects of GSPE on apoptosis markers are dose, time, and/or gender dependent.

## Figures and Tables

**Table 1:** Effects of the cafeteria diet and GSPE treatment on plasmatic glucose and insulin levels, HOMA-IR and HOMA-*β* index. ∗∗*P* ≤ 0.01, ∗∗∗*P* ≤ 0.001, and ^#^ *P* ≤ 0.1 for cafeteria diet versus standard diet. ^‡^ *P* ≤ 0.05 and ^†^ *P* ≤ 0.1 for cafeteria + GSPE versus cafeteria + vehicle.

	Standard diet	Cafeteria diet	Cafeteria + vehicle	Cafeteria + GSPE
Glucose (mM)	4.13 ± 0.2	4.49 ± 0.3	4.76 ± 0.2	4.88 ± 0.2
Insulin (ng/mL)	1.23 ± 0.1	2.47 ± 0.2∗∗∗	2.78 ± 0.2	1.80 ± 0.3^‡^
Insulin/Glucose	6.02 ± 0.6	12.40 ± 1.6∗∗	13.25 ± 0.7	8.39 ± 1.2^‡^
HOMA-IR	4.97 ± 0.5	10.75 ± 1.0∗∗∗	14.66 ± 2.8	8.37 ± 1.2^†^
HOMA-*β*	609.45 ± 93.4	1508.98 ± 388.9^#^	1084.1 ± 13.7	750.58 ± 121.2^‡^

**Table 2:** Effects of cafeteria diet on gene expression in the pancreas. ∗*P* ≤ 0.05 and ^#^ *P* ≤ 0.1 versus standard diet.

	Standard diet	Cafeteria diet
*Ins2 *	1.22 ± 0.3	5.37 ± 1.4∗
*Cpe *	1.16 ± 0.3	3.71 ± 0.8∗
*Bcl-2 *	1.15 ± 0.3	0.28 ± 0.1∗
*Bax *	1.08 ± 0.2	1.64 ± 0.4
*Bcl-2/Bax *	1.27 ± 0.5	0.24 ± 0.1^#^
*Ccnd2 *	1.31 ± 0.4	1.12 ± 0.3

**Table 3:** Effects of GSPE treatment of cafeteria-fed rats on gene expression in the pancreas. ∗∗*P* ≤ 0.01 and ^#^ *P* ≤ 0.1 versus vehicle-treated group.

	Cafeteria + vehicle	Cafeteria + GSPE
*Ins2 *	1.16 ± 0.3	0.55 ± 0.1^#^
*Cpe *	1.04 ± 0.2	0.2 ± 0.1∗∗
*Bcl-2 *	1.12 ± 0.2	0.96 ± 0.3
*Bax *	1.13 ± 0.3	1.18 ± 0.2
*Bcl-2/Bax *	1.67 ± 0.4	0.95 ± 0.4
*Ccnd2 *	1.15 ± 0.3	1.80 ± 0.3
